# HHV-6A Infection of Endometrial Epithelial Cells Induces Increased Endometrial NK Cell-Mediated Cytotoxicity

**DOI:** 10.3389/fmicb.2017.02525

**Published:** 2017-12-15

**Authors:** Elisabetta Caselli, Daria Bortolotti, Roberto Marci, Antonella Rotola, Valentina Gentili, Irene Soffritti, Maria D’Accolti, Giuseppe Lo Monte, Mariangela Sicolo, Isabel Barao, Dario Di Luca, Roberta Rizzo

**Affiliations:** ^1^Section of Microbiology and Medical Genetics, Department of Medical Sciences, University of Ferrara, Ferrara, Italy; ^2^School of Medicine, University of Geneva, Geneva, Switzerland; ^3^Human Reproduction Centre – Brunico Hospital, Brunico, Italy; ^4^School of Medicine, University of Nevada, Reno, NV, United States

**Keywords:** endometrium, HHV-6, NK cell, infertility, epithelial cell

## Abstract

**Background:** We have recently reported the presence of Human herpesvirus-6A (HHV-6A) DNA in the 43% of endometrial epithelial cells from primary idiopathic infertile women, with no positivity in fertile women. To investigate the possible effect of HHV-6A infection in endometrial (e)NK cells functions, we examined activating/inhibitory receptors expressed by eNK cells and the corresponding ligands on endometrial cells during HHV-6A infection.

**Methods:** Endometrial biopsies and uterine flushing samples during the secretory phase were obtained from 20 idiopathic infertile women and twenty fertile women. HHV-6A infection of endometrial epithelial cells was analyzed by Real-Time PCR, immunofluorescence and flow cytometry. eNKs receptors and endometrial ligands expression were evaluated by immunofluorescence and flow cytometry.

**Results:** We observed the presence of HHV-6A infection (DNA, protein) of endometrial epithelial cells in the 40% of idiopathic infertile women. The eNK from all the subgroups expressed high levels of NKG2D and NKG2A receptors. Functional studies showed that NKG2D activating receptor and FasL are involved in the acquired cytotoxic function of eNK cells during HHV-6A infection of endometrial epithelial cells. In the presence of HHV-6A infection, eNK cells increased expression of CCR2, CXCR3 and CX3CR1 chemokine receptors (*p* = 0.01) and endometrial epithelial cells up-modulated the corresponding ligands: MCP1 (Monocyte chemotactic protein 1, CCL2), IP-10 (Interferon gamma-induced protein 10, CXCL10) and Eotaxin-3 (CCL26).

**Conclusion:** Our results, for the first time, showed the implication of eNK cells in controlling HHV-6A endometrial infection and clarify the mechanisms that might be implicated in female idiopathic infertility.

## Introduction

Human herpesvirus 6 (HHV-6) is a ubiquitous pathogen of the *Betaherpesvirinae* subfamily, which primarily infects CD4^+^ T cells (Takahashi et al., 1989). Similarly to other herpesviruses, HHV-6 remains in latency into the host, after an initial productive infection (Sandhoff et al., 1991). HHV-6 is a set of two related viruses known as HHV-6A and HHV-6B (Ablashi et al., 2014). Even if these two viruses present a similar genetical sequence, they differ for biological and pathogenic characteristics. HHV-6B causes exanthema subitum in young children (Yamanishi et al., 1988). HHV-6A seems to be involved in other pathologies, such as multiple sclerosis (Soldan et al., 1997) and encephalitis (McCullers et al., 1995). Moreover, we have recently shown the presence of HHV-6A, but not HHV-6B infection in endometrial epithelial cells of a subgroup of idiopathic infertile women (Marci et al., 2016). HHV-6 infection is implicated in immune-suppressive effects: (i) direct infection and induction of apoptosis of CD4+ T lymphocytes (Lusso et al., 1988; Grivel et al., 2003); (ii) lysis of cytotoxic leukocytes (CD8+ T cells, NK cells) (Lusso et al., 1991; Lusso and Gallo, 1995); (iii) block of dendritic cells and macrophages maturation (Kakimoto et al., 2002; Smith et al., 2005); (iv) inability of macrophages and dendritic cells to produce IL-12p70 after interferon gamma induction (Flamand et al., 1995; Smith et al., 2003, 2005); (v) dysregulation of cytokine networks, with increased secretion of IL-10, RANTES, TNF-alpha and IL-1beta (Flamand et al., 1991); (vi) decreased expression of CD14, CD64 and HLA-DR on the surface of monocytes as a mechanism of immune evasion (Janelle and Flamand, 2006).

Natural killer (NK) cells, positive for the surface marker CD56, are the dominant immune cell type at the uterine mucosa during placentation (Siewiera et al., 2013). They accumulate during implantation, where they support invading placental trophoblast cells and the creation of new vessels, essential for blood supply to the fetus.

The human endometrium contains a substantial population of NK cells (eNK cells) which vary in number and in proportion to the total number of endometrial stromal cells during the menstrual cycle. Although present in proliferative endometrium, eNK cells increase in number substantially in the mid-secretory phase and are the major endometrial lymphocyte population in the late secretory phase and the first trimester of pregnancy. eNK cells are CD56bright CD16+ and also express CD9, which is not expressed by peripheral blood NK cells. In contrast to peripheral blood CD56bright CD16– NK cells, eNK cells have abundant cytoplasmic granules containing perforin and granzyme (Bulmer et al., 1991). There is no consensus about the origin of eNK cells. Mature peripheral blood NK cells or immature precursors may migrate into the endometrium from the blood possibly in response to chemokines produced by cells within the endometrium at specific stages of the menstrual cycle and pregnancy, and be modified by other factors within the endometrium. For example, production of CXCL-12 by extravillous trophoblast (EVT) cells may attract NK cells into the decidua in pregnancy (Wu et al., 2005); interleukin (IL)-15, produced by secretory endometrium and decidua, has a selective chemoattractant effect on peripheral blood CD16– NK cells (Kitaya et al., 2007); and transforming growth factor beta 1 (TGF-1) has been suggested as modifying peripheral blood NK cells to eNK cells (Keskin et al., 2007). An alternative suggestion is that eNK cells are derived from haematopoietic precursor cells within the endometrium (Lynch et al., 2007).

The presence of eNK cells in close proximity to the invading extravillous trophoblast cells suggests that they may play a role in this process. eNK cells produce many different cytokines and growth factors (for example, IL-1, IL-2, IL-4, IL-6, IL-8, IL-10, tumor necrosis factor alpha, granulocyte-macrophage colony stimulating factor, TGF-1, leukemia inhibitory factor and interferon gamma) (Jokhi et al., 1997). eNK cells are also an important source of angiogenic growth factors. Production of angiogenin, angiopoietin (Ang)-1, Ang-2, vascular endothelial growth factor (VEGF)-A, VEGF-C, placental growth factor, keratinocyte growth factor, fibroblast growth factor and platelet-derived growth factor-BB by eNK cells from secretory phase endometrium and early pregnancy decidua has been reported (Li et al., 2001; Lash et al., 2006). eNK cells secrete also matrix metalloproteinases (MMP)-1, MMP-2, MMP-7, MMP-9, MMP-10, tissue inhibitor of metalloproteinases (TIMP)-1, TIMP-2, TIMP-3, urokinase plasminogen activator (uPA) and uPA receptor (Naruse et al., 2009a,b).

The observation of modified percentages of eNK cells in the endometrium of women with reproductive failure (such as infertility, RM and pre-eclampsia) has suggested they may play a role in the pathogenesis. Several studies have shown increased levels of eNK cells in the pre-pregnancy endometrium of women with recurrent miscarriage (Clifford et al., 1999; Tuckerman et al., 2007). There are studies showing an increased number of eNK cells in decidual tissue (Stallmach et al., 1999; Bachmayer et al., 2006) and others showing a reduction in decidual eNK cell numbers in women with pre-eclampsia (Eide et al., 2006; Williams et al., 2009). The presence of a unique type of NK cells in the endometrium is intriguing and the possibility that a microbial infection might affect their functions should be taken into account.

In this article, we provide the first evidence that eNK cells functions are affected by HHV-6A infection of the endometrial epithelial cells and this modification might influence pregnancy outcome.

## Materials and Methods

### Clinical Samples

Endometrial speciments were obtained from patients admitted for tubal patency assessment by Hystero-sono contrast sonography at secretory stage of the menstrual cycle. We selected women with these characteristics: 21–38 years old, regular menstrual cycle (24–35 days), body mass index (BMI) ranging between 18 and 26 Kg/m2, FSH (day 2–3 of the menstrual cycle) <10 mUI/mL, 17-β-Estradiol <50 pg/ml (day 2–3 of the menstrual cycle), normal karyotype. Women that presented endometritis, endometriosis, tubal factor, ovulatory dysfunction, anatomical uterine pathologies and recurrent miscarriage were excluded. Endometrial samples were maintained in HEPES-buffered Dulbecco modified Eagle medium/ Hams F-12 (DMEM/F-12; Invitrogen, Carlsbad, CA, United States) with 1% antibiotic- antimycotic solution (final concentrations: 100 μg/ml penicillin G sodium, 100 μg/ml streptomycin sulfate, 0.25 μg/ml amphotericin B; Invitrogen), and 5% newborn calf serum (NCS; CSL Ltd., Parkville, VIC, Australia), stored at 4°C, and processed within 2 h. Uterine flushing was performed with a 14-gauge Foley three-way balloon catheter (Eschmann) inflating an appropriate (5 mL) amount of sterile physiologic saline solution.

### Ethics Statement

This study was approved by the “Ferrara Ethics Committee” and we collected written informed consent from all subjects. All subjects gave written informed consent in accordance with the Declaration of Helsinki.

### Preparation of Endometrial Epithelial and Stromal Cells

The endometrium was prepared as previously described (Marci et al., 2016). Cell dissociation was performed in Ca2+ and Mg2+ free phosphate buffered saline (PBS, pH 7.4) additionated with 300 μg/ml collagenase type III (Worthington Biochemical Corporation, Freehold, NJ, United States) and 40 μg/ml deoxyribonuclease type I (Roche Diagnostics, Mannheim, Germany) in a shaking incubator (Bioline 4700; Edwards Instrument Company, Narellan, NSW, Australia) rotating at 150 rpm at 37°C. Every 15 min, the digested tissues were omogenized vigorously and dissociation was checked microscopically. After 45 min, the digests were filtered using a 40-μm sieve (Becton Dickinson Labware, Franklin Lakes, NJ, United States) to obtain a single cells suspension without debris. The digestion process was stopped by the addition of HEPES-buffered DMEM/F-12 containing 5%FCS. We isolated the different endometrial cellular components (mononuclear, stromal and epithelial cells) by centrifugation for 8–10 min at 390 × *g* on Ficoll-Paque (Pharmacia Biotechnology, Uppsala, Sweden). Endometrial cells were collected from the Ficoll-Paque-medium interface using BerEP4-coated magnetic Dynabeads (Dynal Biotech, Oslo, Norway) positive selection system. The sorted epithelial cells were recovered using a magnetic particle collector (Dynal Biotech) and washed in HEPES-buffered DMEM/F-12/1%FCS. The collected cells were finally seeded on culture plates coated with basement membrane extract (BME) (Matrigel^®^, Collaborative Biomedical Products, Bedford, MA, United States). The fraction containing mononuclear and stromal cells were collected and seeded on 100 mm plastic tissue culture dishes. After 12 h, the supernatant cells were collected from the culture, to recover non-adherent mononuclear cells. Purity of epithelial and stromal components was morphologically evaluated by light microscopy and assessed by cytokeratin-18 (CK18) and vimentin staining for epithelial and stromal cells respectively. Mononuclear cells were CD45^pos^ stained. The purity reached for each cell population was routinely over 98%.

### DNA Analysis

DNA extraction and analysis were performed as previously described (Caselli et al., 2012). PCR and real time quantitative (qPCR) specific for the U94 gene were used to determine HHV-6 DNA presence and load. Samples in which 1 μg of cell DNA harbored more than 100 copies of viral DNA, were considered positive. Human RNase P or beta-actin house-keeping genes were used as a control. All clinical samples were randomly and blindly investigated. Furthermore, when enough material to repeat the analysis was present, the analysis was repeated again in a randomized and blinded fashion at a distant time from the first analyses. HHV-6A or B identification was performed as reported previously (Caselli et al., 2012), by restriction enzyme digestion of the U31 nested PCR amplification product and visualization of the digestion products on ethidium bromide stained agarose gel after electrophoresis migration.

### RNA Analysis

RNA cell extraction was performed using the RNeasy kit (Qiagen, Hilden, Germany). Extracted RNA did not contain contaminant DNA, as assured by DNase treatment and control β-actin PCR without retrotranscription reverse transcription (Caselli et al., 2012). RNA reverse transcription was performed by the RT2 First strand kit (Qiagen, Hilden, Germany) for analysis of virus transcripts. cDNA aliquots corresponding to 200 ng RNA were used for virus transcription analysis, performed by qPCR detecting the expression of U94 gene, as previously reported (Caselli et al., 2012).

### Immunofluorescence Assay for HHV-6 Detection

HHV-6 antigen expression was analyzed by immunofluorescence with a mouse monoclonal antibodies (mAb) that recognized the glycoprotein gp116 (late antigen) of HHV-6 A and B (ABI, Columbia, MD, United States), as previously described (Marci et al., 2016). Epithelial and stromal cells were respectively stained with mouse anti-Cytokeratin-18, a heterotetramer cytoskeleton protein (CK18-FITC) and rabbit anti-vimentin-PE moAbs (Abcam, Cambridge, United Kingdom), respectively. eNK cells were stained for CD56-PE (BD, Milan, Italy). Epithelial cells were stained for IP-10-FITC (Interferon gamma-induced protein 10/CXCL10), Eotaxin-3-PE (CCL26) and MCP-1-PE (Monocyte chemo-attractant protein 1/CCL2) (eBiosciences, Waltham, MA, United States).

### NK Cell Purification

Endometrial NK cells were separated from endometrial leukocyte samples using the negative magnetic cell separation (MACS) system (Miltenyi Biotech, Gladbach, Germany) (Marci et al., 2016). The analysis of purified cell fraction by flow cytometry with CD3-PerCp-Cy5.5, CD56-FITC moAbs (e-Bioscience, Frankfurt, DE), demonstrated that the NK cell content was >90% (data not shown). Freshly purified NK cells were cultured for 24 h in presence of suboptimal doses of IL-12 (1 ng/ml).

### HHV-6A Cell Infection

Primary endometrial cells and KLE endometrial epithelial cell line (ATCC CRL1622) were cultivated in DMEM F12 medium (ATCC 30-2006) in presence of L-glutamine, 1% penicillin-streptomycine and 10% of FCS at 37°C with the 5% of CO2. The cells were inoculated with HHV-6A (strain U1102) cell-free virus inocula (Marci et al., 2016) and infected with 100 genome equivalents per 1 cell. HHV-6A UV-inactivated viral preparations were used as controls. Infected cells were co-cultured with eNK cells to perform cytotoxicity experiments.

### Cytotoxicity Assays

Target cells (1 × 10^6^ cells) were labeled with 7-AAD/CFSE Cell-Mediated Cytotoxicity Assay Kit (Cayman Chemicals, Ann Arbor, MI, United States), that employs CFSE to label target cells within the mixed cell population and 7-AAD to label dead cells. We added eNK effector cells to labeled target cells at various effector:target ratios in replicate in 200 ml DMEM/F12 containing 5% FCS. Microtiter plates were centrifuged at 1200 rpm for 5 min and incubated at 37°C. After 4 or 18 h of culture, the cells were analyzed by flow cytometry.

To block activating receptor engagement or FASL/FAS pathway, CFSE labeled target cells were incubated with soluble NKG2D-Fc IgG1 chimeric protein (0.2 mg/ml) (R&D Systems, Italy) and/or anti-FASL mAb (10 mg/ml; R&D Systems, Italy) or an isotype match control (1.0 mg/ml; mouse IgG1) for 20 min on ice then co-cultured with eNK effector cells. eNK cell cytotoxicity against K562 (ATCC CCL243), classical target cell, was used as control.

### ELISA sFASL, sTRAIL, MCP1, IP10, Eotaxin3

Levels of sTRAIL, sFasL, MCP1, IP10 and Eotaxin3 were assessed in duplicates in cell culture supernatants and uterine flushing samples using commercial enzyme-linked immunosorbent assays (ELISAs) (TRAIL, MCP1, IP10, Eotaxin3: R&D Systems, Amersham, United Kingdom; sFasL: Sigma–Aldrich, St. Louis, MO, United States).

### Flow Cytometry

Leukocytes were defined as CD45pos, and the different cell subtypes were defined as CD3pos (T cells), CD19pos (B cells) CD14pos (monocytes) and Cd56pos (NK cells). Cell viability was assessed by propidium iodide staining. Anti-isotype controls (Exbio, Praha, CZ) were performed.

1 × 10^5^ eNK cells were labeled with fluorophore-conjugated antibodies: CD3-PE-Cy7, CD16-PE, CD56-APC, NKG2D-PE, NKG2C-PE, NKG2A-PE, NKp30-PE, NKp44-PE, NKp46-PE, KIR2DL2/3-PE, KIR2DL1-PE (BD, Italy), KIR2DL4-PE (R&D Systems, Italy); CXCR1, CXCR2, CXCR3, CX3CR1, CXCR4, CCR1, CCR2, CCR3, CCR5, and CCR7 (R&D Systems, Italy). eNK cells were gated as CD56pos CD3neg. eNK cells activation status was determined by CD107a staining (R&D Systems, Italy), as previously reported (Rizzo et al., 2016).

5 × 10^5^ epithelial cells were stained specific Ab HLA-I (HLA-A,-B,-C)-PE (BD Biosciences, Italy), HLA-E (clone MEM-E/08, Exbio, Praha, CZ) or HLA-DR (BD Biosciences, Italy) and matched isotype controls.

The NKG2D-ligands were detected on epithelial cells by binding of NKG2D-Fc chimera (R&D Systems, Italy) and indirect labeling with the secondary Ab FITC-coupled mouse anti-human IgG1 (Abcam, Cambridge, United Kingdom).

Data were analyzed using FACS CantoII flow cytometer (BD, Milan, Italy) and FlowJo LLC analysis software (Ashland, OR, United States). Ten thousand events were acquired.

### Statistical Analysis

Data were analyzed by Student’s *t*-test and Fisher exact test [Stat View software (SAS Institute Inc.)]. Statistical significance was assumed for *p* < 0.05 (two tailed).

## Results

### HHV-6 in Clinical Specimens

We enrolled 20 idiopathic infertile women and 20 fertile women with at least one previous successful pregnancy. As reported in **Table 1**, the two cohorts presented no significant differences.

**Table 1:** Women cohorts: demographical and clinical parameters.

Parameters (median; mean ± SD)	Infertile (20)	Fertile (20)	*p*-value^∗^
Age (years)	34.4 (32.2 ± 2.2)	33.6 (31.5 ± 2.9)	0.95
Duration of infertility (years)	3.2 (3.7 ± 1.9)	-	-
Length of menstrual cycle (days)	3.9 (3.8 ± 1.9)	4.1 (3.7 ± 2.5)	0.54
FSH (mUI/mL) (day 3)	7.9 (8.2 ± 1.7)	7.6 (7.4 ± 2.9)	0.53
LH (mUI/mL) (day 3)	6.8 (6.6 ± 2.5)	7.0 (6.8 ± 2.4)	0.51
Estradiol (pg/mL) (day 3)	71.6 (72.1 ± 63.5)	66.4 (58.2 ± 49.2)	0.052
TSH (uUI/mL)	3.1 (2.8 ± 1.5)	2.8 (2.6 ± 1.4)	0.48
FT4 (pg/mL)	2.4 (2.3 ± 1.2)	2.5 (2.4 ± 1.4)	0.91
Progesterone (pg/mL) (day 21)	15.9 (14.7 ± 6.2)	14.6 (13.9 ± 6.4)	0.72
Smoke habits (%)^∗∗^	10	9.6	0.92
Day (mestrual cycle) of sample collection	14.1 (13.8 ± 2.6)	14.0 (13.2 ± 2.8)	0.98

*^∗^Student’s *t*-test. ^∗∗^Fisher exact test.*

The endometrial biopsies were analyzed for the presence of HHV-6 infection. As reported in **Table 2**, we found HHV-6B DNA in the peripheral blood mononuclear cells of the 24 and 26% of idiopathic infertile women and control women, respectively (*p* = 0.86; Fisher exact test). These results confirmed the previously reported frequency of the HHV-6B virus in a 25–30% of peripheral blood samples (Caselli and Di Luca, 2007). On the contrary, HHV-6A DNA was not revealed in the peripheral blood of all the subjects. Forty percent (8/20) women with idiopathic infertility were positive for HHV-6 DNA in their endometrial epithelial cells, while fertile women did not present HHV-6A viral DNA in their endometrial epithelial cells (*p* = 4.5 × 10^-6^; Fisher exact test). HHV-6B DNA was not present in all the endometrial biopsies, as previously reported (Marci et al., 2016).

**Table 2:** HHV-6 DNA results in peripheral blood mononuclear cells (PBMC) and endometrial biopsies.

Samples (*N*)	Infertile (20)	Fertile (20)	*p*-value^∗^
HHV6-A			
Endometrial epithelium	8	0	0,003
Endometrial stroma	0	0	NA
Endometrial leukocytes (CD45pos)	0	0	NA
PBMC	0	0	NA
HHV-6B			
Endometrial epithelium	0	0	NA
Endometrial stroma	0	0	NA
Endometrial leukocytes (CD45pos)	0	0	NA
PBMC	5	6	0,86

*^∗^Fisher exact test.*

**FIGURE 1:**
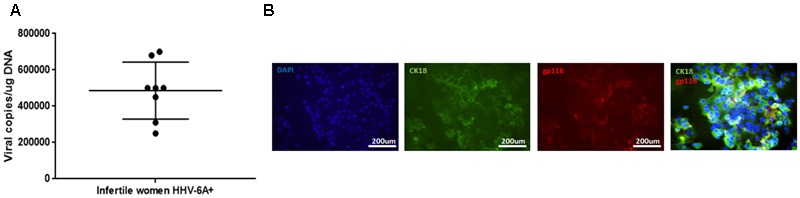
HHV-6A infection of endometrial epithelial cells. **(A)** HHV-6 DNA was searched by real time qPCR specific for U94 gene in endometrial biopsies. Results are expressed in viral copies/ug DNA and represent the mean copy number ± SD referred to duplicates of 2 independent assays. Infertile women: primary idiopathic infertile women; **(B)** Endometrial epithelial cells were characterized by immunofluorescence for cytokeratin18 (CK18, epithelial marker) and gp116 (late viral protein) expression. Images were taken in fluorescence (Nikon Eclipse TE2000S) equipped with a digital camera. Original magnification 20×.

The average viral load in endometrial epithelial cells (vimentin^neg^ CK18^pos^; Supplementary Figure S1A) from HHV6-A positive infertile women was 500.000 copies/ug of cellular DNA (range 700.000–240.000 copies/μg DNA), corresponding to about 4 copies of viral DNA per diploid cell (**Figure 1A**). Endometrial epithelial cells were HHV-6A positive as shown by staining for gp116 late viral antigen (**Figure 1B**). Stromal cells (Vimentin^pos^ CK18^neg^; Supplementary Figure S1B) and leukocytes (CD45^pos^; Supplementary Figure S1C) were negative for HHV-6 DNA (**Table 2**), confirming a localized HHV-6A infection into endometrial epithelium, as previously reported (Marci et al., 2016).

### Endometrial Epithelial Cells Are Permissive to HHV-6A Infection

**FIGURE 2:**
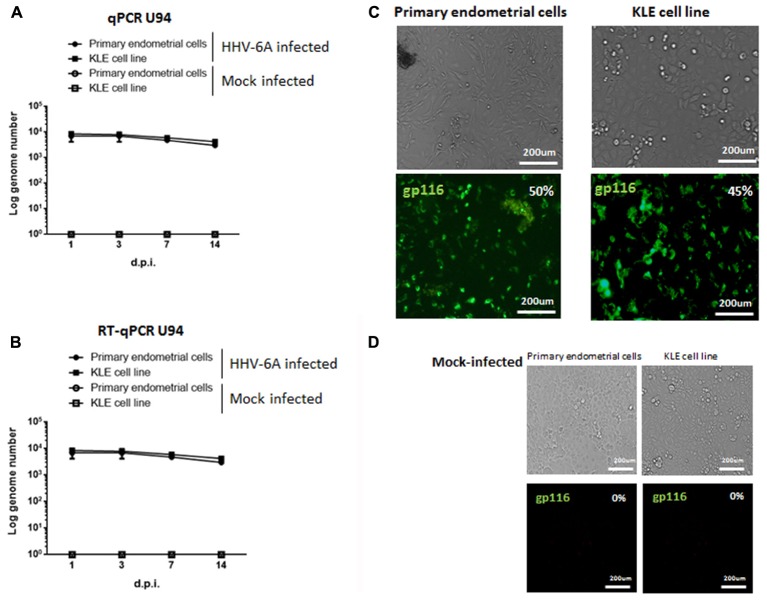
Primary endometrial epithelial cells and KLE cell line (ATCC CRL-1622) were infected with HHV-6A or UV-inactivated (Mock-infected) HHV-6A. The infection was evaluated after 1, 3, 7, and 14 days post infection (d.p.i.). **(A)** DNA and **(B)** RNA expression of viral U94 antigen were evaluated by Real time PCR. Results represent the mean copy number ± SD referred to duplicates of 4 independent assays. **(C,D)** Endometrial epithelial cells were stained for gp116 late viral antigen 7 d.p.i. with HHV-6A **(C)** or mock **(D)**. Images were taken in bright field (*upper panels*) or fluorescence (*lower panels*) (Nikon Eclipse TE2000S) equipped with a digital camera. Original magnification 20×.

Primary endometrial epithelial cells, purified from endometrial biopsies from fertile women negative for HHV-6A infection (Vimentin^neg^ and CK18^pos^, Supplementary Figure S1), and the KLE endometrial epithelial cell line were infected with HHV-6A cell-free inoculum with 100 genome equivalents per cell. Mock infection with UV-inactivated virus was used as control. We evaluated viral replication 1, 3, 7, and 14 days post-infection (d.p.i.) by analyzing virus DNA, transcription and antigen expression. As shown in **Figure 2A**, HHV-6A DNA was present at all times p.i. Virus load, as evaluated by real-time quantitative PCR in primary endometrial epithelial cells and KLE cell line, respectively, corresponded to 7.0 × 10^4^ and 8.1 × 10^4^ genome copies per μg of total cellular DNA at 1 d.p.i., 5.4 × 10^4^ and 6.2 × 10^4^ at 3 d.p.i. and 3.2 × 10^4^ and 4.5 × 10^4^ at 14 d.p.i., when the experiment was discontinued. Virus U94 mRNA, an immediate early gene, was analyzed to establish viral transcription (Pantry and Medveczky, 2017). We observed the persistence of U94 mRNA in all time points (**Figure 2B**). The late HHV-6 gp116 antigen expression confirmed that both primary and KLE endometrial epithelial cells were permissive to HHV-6A replication. gp116 virus antigen expression, indicative of productive infection, peaked at 7 d.p.i., (**Figure 2C**). Control mock-infected endometrial epithelial cells gave no staining (**Figure 2D**).

We previously showed that HHV-6A positive women have an increased cytolytic function of eNK cells toward HHV-6A infected cells (Marci et al., 2016), suggesting an implication of viral infections in the modulation of eNK cell functions. Therefore, to test the ability of eNK cells in controlling HHV-6A infection, we analyzed their cytotoxicity against HHV-6A-infected endometrial epithelial cells, by 7-AAD/CFSE Cell-Mediated Cytotoxicity Assay Kit.

**FIGURE 3:**
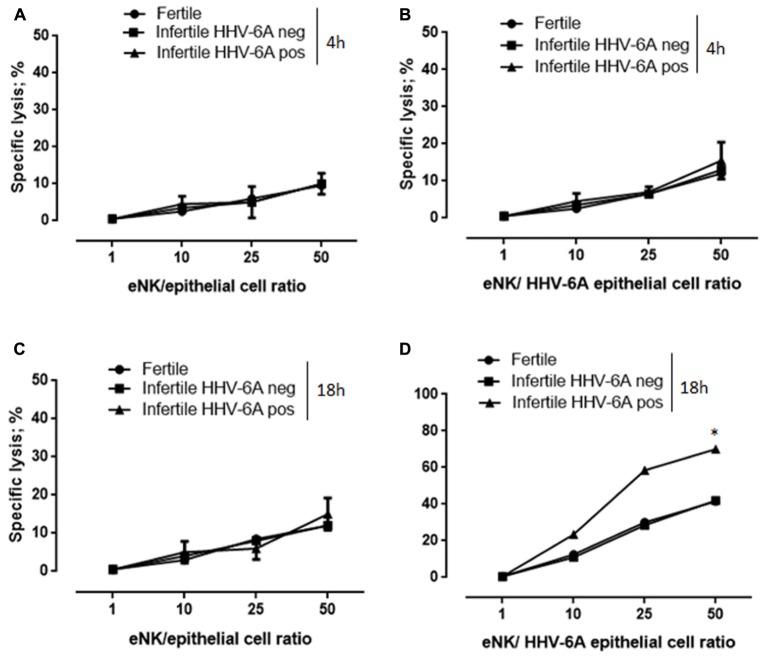
eNK cells are cytotoxic against HHV-6A-infected endometrial epithelial cells. Endometrial epithelial cells were kept **(A,C)** uninfected or **(B,D)** infected for-48 h with HHV-6A. eNK cell cytotoxicity was determined by 7-AAD/CFSE Cell-Mediated Cytotoxicity Assay Kit after **(A,B)** 4 or **(C,D)** 18 h of contact at different E/T ratios. Each data point is calculated as the mean lysis ± SD from at least five independent experiments done in replicate tissue culture wells. ^∗^Significant *p*-value; Student’s *t*-test.

The co-culture of eNK cells with HHV-6A infected endometrial for 4 h did not result in an efficient killing (**Figure 3B**). However, after 18 h of co-culture, eNK cells killed HHV-6A infected endometrial epithelial cells (**Figure 3D**). With eNK cells from HHV-6A positive women at the effector:target ratio of 50 we observed up to 75% of killing, while only 40% of killing was obtained with eNK cells from HHV-6A negative women (*p* < 0.001; Student’s *t*-test). The absence of killing of uninfected endometrial epithelial cells, supports the specificity of the eNK cytotoxic function against HHV-6A infection (**Figures 3A,C**). Taken together, these results suggest that in the presence of HHV-6A infection eNK cells from HHV-6A positive women become more cytotoxic against infected endometrial epithelial cells. To exclude the presence of external factors that could modify the eNK cell cytotoxicity in HHV-6A positive women, we tested the killing of K562 cells, a classical NK cell targets. In agreement with previous studies (Kopcow et al., 2005), we observed a low cytotoxicity of eNK cells in the presence of K562 cells in both HHV-6A positive and negative women settings (Supplementary Figures S2A,B). These results suggest a peculiar modification of eNK cells in the presence of HHV-6A-infection, that seems to induce the loss of their tolerance toward endometrial epithelial cells. This condition is enhanced in HHV-6A positive women.

### NKG2D Receptor Modulates eNK Cell Responsiveness to HHV-6A-Infected Endometrial Epithelial Cells

NK cells cytotoxicity is controlled by several NKRs. To evaluate their possible implication in eNK cell cytotoxicity against HHV-6A-infected endometrial epithelial cells, we analyzed the expression of activating/inhibitory receptors in eNK cells.

**FIGURE 4:**
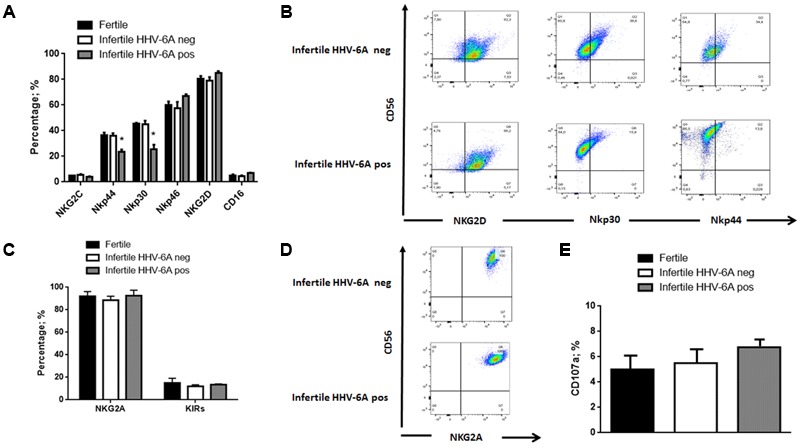
eNK cell receptor repertoire expression. eNK cells purified from endometrial biopsies were stained for surface expression of the indicated **(A)** activating and **(C)** inhibitory receptors and **(E)** CD107a activation marker using fluorochrome-conjugated antibodies and analyzed by flow cytometry. **(B,D)** Representative FACS dot plots gated on CD56pos CD3neg dNK cells are reported. One representative dot plot out of five independent experiments is shown. ^∗^Significant *p*-value obtained by Student’s *t*-test.

eNK cells, gated as CD3^neg^ and CD56^pos^, were purified from endometrial biopsies from fertile and infertile women. Flow cytometry analysis showed that eNK cells express NKG2D, but express low levels of the activating receptors NKG2C/CD94 and CD16 (**Figure 4A**). Even though the activating receptors NKp44, and NKp30 are expressed in all eNK cells (**Figure 4A**), there is a decreased expression in infertile women positive for HHV-6A infection (**Figures 4A,B**) (*p* < 0.001; Student’s *t*-test). This difference in expression pattern makes eNK cells from infertile women positive for HHV-6A infection quite different from eNK cells commonly present in endometrial tissues (ref J Immunol, **Figure 4**), and from decidual NK cells, which express relatively high levels of NKp30, NKp44, and NKG2D (Hanna et al., 2006).

Expression of inhibitory receptors showed that all eNK cells express high levels of NKG2A/CD94 receptor and low expression of KIRs (Killer immunoglobulin like receptors) (**Figures 4C,D**).

These results demonstrate a possible role of HHV-6A infection in changing in eNK cell receptor repertoire, decreasing NKp30 and NKp44 and maintaining NKG2D and NKG2A expression.

The expression of inhibitory and activating receptors is implicated in the immunoregulation of eNK cells functions to control the uterine environment and embryo implantation. A modification of receptors ligands caused by HHV-6A infection could undermine eNK cells peculiar functions.

We analyzed activation status of eNK cells, measuring CD107a expression, that correlates with both cytokine secretion and NK cell-mediated lysis of target cells (Alter et al., 2004). We observed a slight but not significant increase in the percentage of eNK positive for CD107a expression in HHV-6A positive infertile women (*p* = 0.08; Student’s *t*-test) (**Figure 4E**).

These results suggest that contact of eNK cells with HHV-6A infected cells is detrimental to recognize differences in eNK cells status. For this, we selected to analyze the effect of HHV-6A infection on ligands for the two NK receptors that maintained a high expression in eNK cells: NKG2D and NKG2A.

**FIGURE 5:**
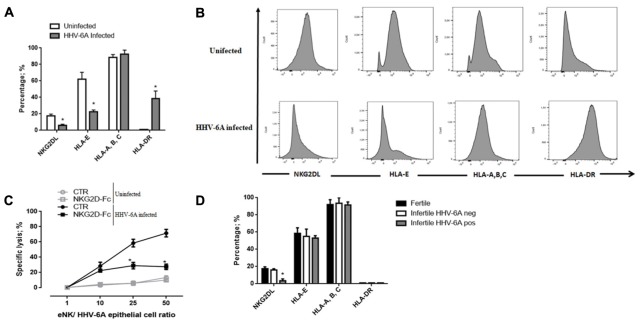
Functional analysis dNK cells specific receptors. **(A,B)** HHV-6A-infection modulates the expression of NKR ligands on endometrial epithelial cells. The binding of human NKG2D-Fc chimera was used to evaluate the cell surface expression of specific receptor ligands. The expression of HLA-E and HLA-A, -B, -C and HLA-DR molecules was evaluated using specific mAb. **(C)** Endometrial epithelial cells uninfected (gray lines) or infected (black lines) were incubated with soluble NKG2D-Fc fusion protein at the concentration of 1 mg/ml and eNK cell cytotoxicity was analyzed by 7-AAD/CFSE Cell- Mediated Cytotoxicity Assay Kit after 18 h of co-culture. **(D)** The expression of NKG2D ligands, NKG2A ligands (HLA-E and HLA-A, -B, -C) and HLA-DR expression on the surface of endometrial epithelial cells from fertile women and HHV-6A positive and negative infertile women. Data sets represent mean lysis ± SD from five independent experiments done in replicate. ^∗^Significant *p*-value; Student’s *t*-test.

Because the identities of the cellular ligands of the activating receptor NKG2D is largely unknown, we stained the endometrial epithelial cells with Ig fusion proteins composed of the extracellular portions of NKG2D fused to human IgG1. NKG2D ligands were down-modulated following infection (**Figures 5A,B**). These results suggest the implication of NKG2D activating receptor in the cytotoxic function of eNK cells toward HHV-6A infected endometrial epithelial cells.

We then analyzed the effect of HHV-6A infection and the expression level of HLA-E molecule, the ligand of inhibitory receptor NKG2A. As shown in **Figures 5A,B**, we observed the expression of both the non-classical HLA-E and the classical HLA-A,-B,-C molecules at the surface of endometrial epithelial cells. The infection with HHV-6A reduced HLA-E expression, while no effect was observed for classical HLA-A, -B, -C. This downregulation of HLA-E expression by HHV-6A excluded any effect of NKG2A inhibitory receptor in controlling eNK cell responsiveness to HHV-6A-infected endometrial epithelial cells. HLA-II antigens are not expressed by endometrial epithelial cells, while they are up-modulated during HHV-6A infection, similar to previously reported the effect on thyrocytes (Caselli et al., 2012) (**Figures 5A,B**).

To further confirm that the cytotoxic function of eNK cells is associated to the NKG2D activating receptor, we next investigated lytic capacities of eNK cells from HHV-6A positive women blocking specific receptor/ligand interactions with a Fc-chimeric protein. The addition of NKG2D Fc-chimeric protein decreased eNK cell cytotoxicity resulting in a 20% lysis of infected cells compared to a 60% lysis of HHV-6A-infected cells, that we observed without the block of receptor/ligand interactions (**Figure 5C**) (*p* < 0.001, Student’s *t*-test). The reduced eNK cytotoxicity in NKG2D ligation blocking experiments sustains a role for NKG2D receptor in eNK cell cytotoxicity.

When we looked at NKG2D and NKG2A ligands expression by endometrial cells from infertile HHV-6A positive and negative women and fertile women, we observed a slight reduction of HLA-E and a significant decrease in NKG2D ligands expression in HHV-6A positive infertile women (**Figure 5D**) (*p* < 0.001, Student’s *t*-test).

### Cytotoxicity of eNK Cells Is Dependent of FasL Killing Pathway

**FIGURE 6:**
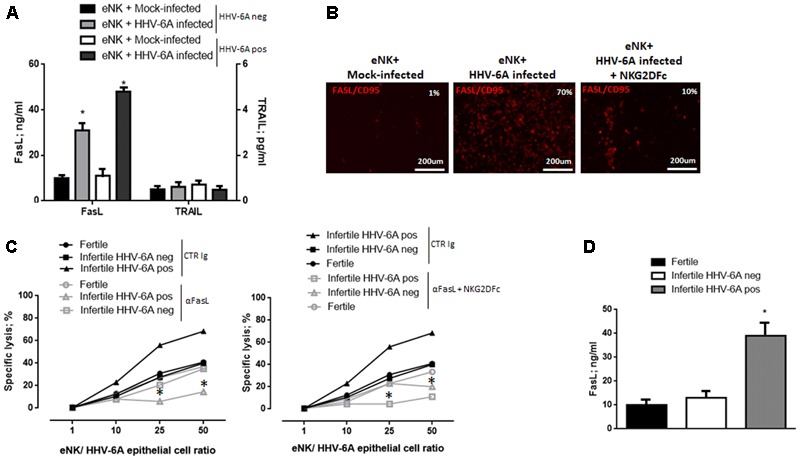
eNK cell cytotoxicity via FasL-Fas pathway. **(A)** Expression of FasL and TRAIL in the culture supernatants of 18 h co-culture of HHV-6A infected endometrial epithelial cells with eNK cells from HHV-6A positive and HHV-6A negative infertile women. **(B)** Expression of FasL in eNK cells after 18 h co-culture with HHV-6A infected endometrial epithelial cells. Images were taken in fluorescence (Nikon Eclipse TE2000S) equipped with a digital camera. Original magnification 20×. **(C)** Functional analysis of FasL. Endometrial epithelial cells were infected for 48 h with HHV-6A. eNK cell cytotoxicity was determined by 7-AAD/CFSE Cell- Mediated Cytotoxicity Assay Kit after 18 h of contact at different E/T ratios, with the addition of a control Ig (CTR Ig; black lines) or of a specific anti FasL Ig (aFasL; gray lines) (left panel) or a specific anti FasL Ig and NKG2DFc (aFasL + NKG2DFc; gray lines). Each data point is calculated as the mean lysis ± SD from at least five independent experiments done in replicate tissue culture wells. **(D)** Expression of FasL in the uterine flushing samples from fertile women and HHV-6A positive and HHV-6A negative infertile women. ^∗^Significant *p*-value; Student’s *t*-test.

NK cells cytotoxicity is mediated by soluble mediators or by induction of death receptor-ligand pathways such as TRAIL (tumor necrosis factor-related apoptosis-inducing ligand) and Fas ligand (FasL). To evaluate the mechanisms involved in eNK cell killing of HHV-6A-infected endometrial epithelial cells, we analyzed these death receptor-ligand pathways. After 18 h of co-culture, FasL (*p* < 0.001; Student’s *t*-test), and not TRAIL, was up-modulated on the surface of eNK cells (**Figures 6A,B**). The blockade of FasL with a neutralizing antibody to FasL reduced eNK cell cytotoxicity against HHV-6A-infected endometrial epithelial cells, after 18 h of co-culture (**Figure 6C**). The blockade of NKG2D recognition of its ligands reduced the expression of FasL on eNK cells (*p* < 0.03; Student’s *t*-test) and the co-blockade of FasL and NKG2D ligands abolished eNK cells killing of HHV-6A-infected endometrial epithelial cells (*p* < 0.02; Student’s *t*-test). These data suggest that NKG2D ligands recognition and mechanisms dependent of the death receptor-ligand Fas/FasL pathway are at the basis of eNK cell killing of HHV-6A infected endometrial epithelial cells.

When we looked at FasL expression by endometrial cells from infertile HHV-6A positive and negative women and fertile women, we observed increased levels of FasL secretion in the uterine flushing samples of infertile HHV-6A positive women (**Figure 6D**) (*p* < 0.001, Student’s *t*-test).

### HHV-6A Infection Modulates the Repertoire of Chemokine Receptors and Their Ligands

The repertoire of ligand to chemokines receptors plays a critical role in eNK cells homing to infected cells. To evaluate the mechanisms implicated in eNK cell cytotoxicity against HHV-6A infected endometrial epithelial cells, we analyzed the expression of chemokine receptors and ligands on eNK cells in the presence or in the absence of HHV-6A infection.

**FIGURE 7:**
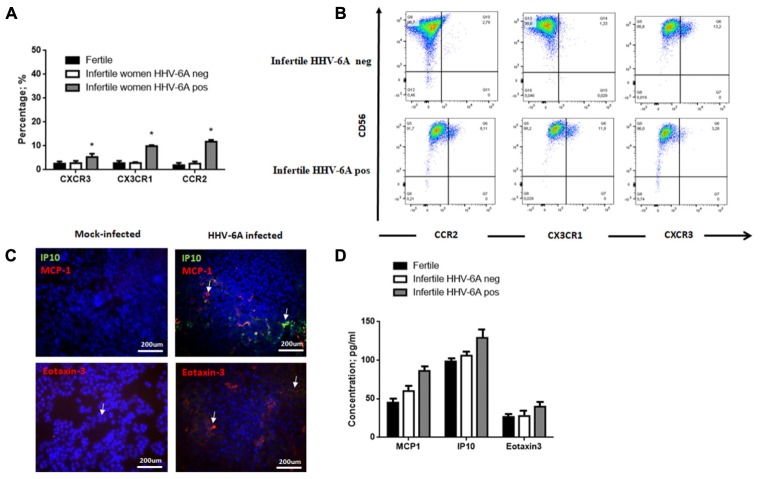
eNK cell receptor repertoire expression. **(A)** eNK cells purified from endometrial biopsies were stained for surface expression of the indicated chemokine receptors using fluorochrome-conjugated antibodies and analyzed by flow cytometry. **(B)** Representative FACS dot plots gated on CD56pos CD3neg dNK cells are reported. One representative dot plot out of five independent experiments is shown. ^∗^Significant *p*-value obtained by Student’s *t*-test. **(C)** Chemokine receptor ligand expression. MCP1 (Monocyte chemotactic protein 1, CCL2), IP-10 (Interferon gamma-induced protein 10, CXCL10) and Eotaxin-3 (CCL26) expression was evaluated in endometrial epithelial cells 7 d.p.i. White arrows indicate positive staining. Images were taken in fluorescence (Nikon Eclipse TE2000S) equipped with a digital camera. Original magnification 20×. **(D)** Chemokine receptor ligand expression. MCP1 (Monocyte chemotactic protein 1, CCL2), IP-10 (Interferon gamma-induced protein 10, CXCL10) and Eotaxin-3 (CCL26) expression was evaluated in uterine flushing samples from HHV-6A positive and negative infertile women and fertile women. ^∗^Significant *p*-value obtained by Student’s *t*-test.

Flow cytometry analysis showed that eNK cells express very low or undetectable levels of CXCR1, CXCR2, CXCR3, CXCR4, CCR1, CCR2, CCR3, CCR5, CCR7 and CX3CR1 (data not shown). We observed a slight increase CXCR3, CX3CR1 and CCR2 receptors in eNK cells from infertile women positive for HHV-6A infection (**Figures 7A,B**) (*p* = 0.02; *p* = 0.012; *p* = 0.01, respectively; Student’s *t*-test).

Meanwhile, we observed an induction of MCP1, IP10 and eotaxin-3 expression on the surface of endometrial epithelial cells 7 d.p.i. (**Figure 7C**). These molecules are the ligands for CCR2, CXCR3 and CX3CR1, respectively, that are upmodulated on eNK cells from HHV-6A positive women (**Figures 7A,B**).

When we analyzed uterine flushing samples for the expression of MCP1, IP10 and eotaxin-3, we observed increased levels in HHV-6A positive infertile women in comparison to HHV-6A negative infertile women and fertile women, with IP10 reaching a significant difference (*p* = 0.031; Student’s *t*-test) (**Figure 7D**).

**FIGURE 8:**
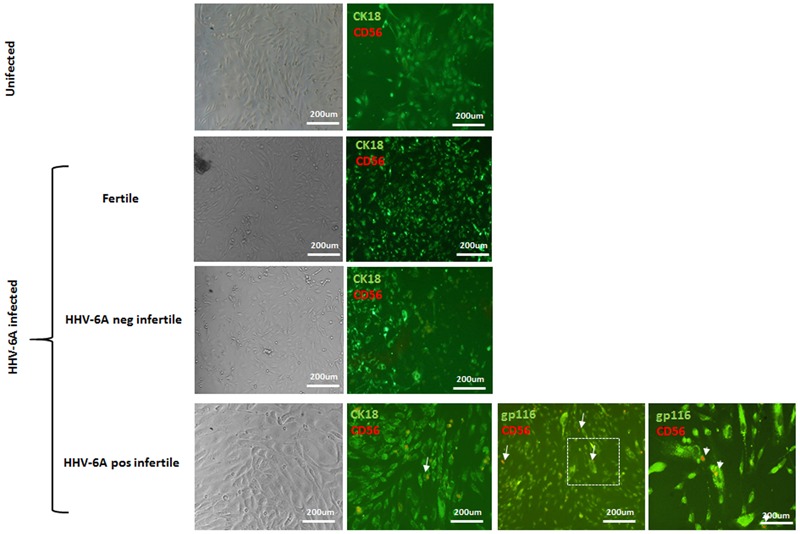
Endometrial epithelial cells were infected for 72 h with HHV-6A. eNK cell were co-cultured for 4 h at a E/T ratio of 10. Endometrial epithelial cells were stained with CK18 (green), eNK cells with CD56 (red) and HHV-6A with gp116 (green). Images were taken in bright field *left panels*) or fluorescence (*right panels*) (Nikon Eclipse TE2000S) equipped with a digital camera. Original magnification 20× and 100×.

Since eNK cells are in close contact with endometrial epithelial cells, we used an *in vitro* culture model of endometrial epithelial cells to test the ability of eNK cells to get in contact with HHV-6A infected endometrial epithelial cells. Endometrial epithelial cells were infected (72 h) or not and co-cultured with eNK cells from HHV-6A positive infertile women for 2 h. As shown in **Figure 8** eNK cells (CD56^pos^) were able to establish contact with HHV-6A infected endometrial epithelial cells but not with uninfected cells. On the contrary, eNK cells from HHV-6A negative infertile and fertile women were not able to interact with HHV-6A infected endometrial epithelial cells.

Together these results evidence the ability of eNK cells to contact HHV-6A-infected endometrial cells, demonstrating the role of eNK cells in controlling HHV-6A infection in the endometrial tissue.

## Discussion

Our study demonstrates, for the first time, the critical role of eNK cells in counteracting HHV-6A infection in endometrial tissues through cytotoxicity. We showed phenotypic and cellular changes in eNK cells that allow the recognition and killing of HHV-6A-infected cells in a FasL-dependent manner.

We demonstrate that under HHV-6A infectious conditions, a significant fraction of eNK cells rapidly dampened down their Nkp30 and Nkp44 expression level, maintaining NKG2D and NKG2A expression.

We further show that control of HHV-6A infection involves NKG2D receptor and that endometrial epithelial cells decrease NKG2D ligands upon HHV-6A infection. This is true also *in vivo*, where endometrial epithelial cells from HHV-6A positive infertile women decreased the expression of NKG2D ligand. These findings suggest that also HHV-6A, as previously reported for HHV-6B (Schmiedel et al., 2016), modifies the expression of activating NKG2D receptor ligands. It should be noted that we obtained discrepant results with a decrease of NKG2DL expression by endometrial cells and the acquisition of cytotoxic effector functions through NKG2D receptor in eNK cells. We hypothesize a selective decrease in NKG2DL with the maintenance of high affinity ligands. Alternatively, co-engagement of other activating receptors could be enough even if there is less NKG2D ligands. Otherwise, the recruitment of NKG2DL into cholesterol-enriched membrane lipid microdomains may promote their shedding, maintaining their functional activity (Waldhauer et al., 2008; Aguera-Gonzalez et al., 2011). The identification of receptor-ligand interactions sustaining eNK cellular cytotoxicity might help to identify potential therapeutic targets that could limit HHV-6A spreading in endometrial tissues. In fact, it has been previously suggested that NKG2D receptor expressed by a subset of virus-specific lymphocytes might behave as a prototypic costimulatory receptor with a role in the control of viral infection in HLA class II^pos^ NKG2DL^pos^ cells, as we observed in endometrial epithelial cells (Saez-Borderias et al., 2006).

The engagement of NKG2D might trigger cytokine production (i.e., IFN-type 1, IFN-gamma, TNF-alpha) that control cytotoxicity of eNK cells by FasL-Fas pathway (Nguyen et al., 2002). We observed the up-regulation of FasL on the surface of eNK cells and in the uterine flushing samples of HHV-6A positive infertile women, suggesting that eNK cell killing of HHV-6A infected endometrial epithelial cells proceeds through NKG2D engagement and subsequent mechanisms dependent on the death receptor-ligand Fas/FasL pathway. Interestingly, both eNK cells from fertile and infertile HHV-6 negative women presented an increase in specific lysis when co-cultured with HHV-6A infected endometrial cells. However, eNK cells from fertile and infertile HHV-6 negative women present a lower lysis that is similar to that obtained when they are co-cultured with K562 target cells (Supplementary Figure S2). For this, we concluded that the lysis obtained with eNK cells from fertile or infertile HHV-6 negative women is due to the natural cytotoxicity of eNK cells in the presence of target cells. On the contrary, eNK cells from HHV-6A positive infertile women showed a significantly higher lysis of HHV-6A infected endometrial cells, that overcomes the natural cytotoxicity of eNK cells.

Similarly, HLA-E molecules, the ligand of the inhibitory NKG2A receptor, are down-modulated during HHV-6A infection and slightly reduced in endometrial epithelial cells from HHV-6A positive infertile women, thus promoting an activatory profile. On the contrary, classical HLA class I molecules maintain their level of expression during HHV-6A infection. The difference between HLA-E and classical HLA-I molecules might reside in their intrinsic functional differences, where HLA-E has an immune-regulatory function while classical HLA-I antigens are totally involved in antigen presentation (Rizzo et al., 2017). HHV-6A might interfere with HLA-E surface expression impairing protein translocation from endoplasmic reticulum. Endometrial epithelial cells acquired a *de novo* expression of HLA-II DR molecules, as previously reported for thyrocytes (Caselli et al., 2012). This “APC-like” phenotype during HHV-6A infection, might be involved in viral clearance by immune cells. The expression of HLA-II antigens and chemokine receptor ligands could facilitate eNK cells homing to HHV-6A infected endometrial cells, as we demonstrated in *in vitro* culture model of endometrial epithelial cells. Since no evidence of HLA-II up-modulation in endometrial epithelial cells from HHV-6A positive infertile women was observed, further investigations are needed to demonstrate the role of HLA-II molecules expression in HHV-6A antigen presentation.

We demonstrate that during HHV-6A infection, eNK cells seem to become cytotoxic to limit viral infection. We observed phenotipical and functional modifications of both eNK cells and endometrial epithelial cells in HHV-6A positive infertile women samples, suggesting an imprint due to HHV-6A infection on both eNK cell immune-phenotype and receptor repertoire toward a cytotoxic activity. Meanwhile, the down-modulation of NKG2D ligands on endometrial epithelial cells could maintain *in vivo* eNK cells with a low killer profile, allowing the persistence of a subclinical HHV-6A infection. We demonstrate that NKG2D activating and chemokine receptors are implicated in eNK cell cytotoxicity toward HHV-6A-infected endometrial cells. The ability of eNK cells to “recognize” HHV-6A infected endometrial cells *in vitro* support the implication of eNK cells in controlling HHV-6A infection and spreading in endometrial environment. To our knowledge, this is the first time evidence for the involvement of eNK cells in controlling HHV-6A endometrial infection. The persistence of activated eNK and of subclinical HHV-6A infection could alter endometrial environment, as demonstrated by the increase in chemokines, mainly IP10, and FasL in uterine flushing samples from HHV-6A positive infertile women. This perturbation of molecular environment might disadvantage embryo implantation and placentation, which require a correct engagement of eNK cells. The presence of activated eNK cells can potentially have serious adverse side effects, as incorrect or insufficient remodeling of the spiral arteries leading to complications of pregnancy such as pre-eclampsia, fetal growth restriction and stillbirth. Nowadays, there are treatments for the control of NK cells activation, as prednisolone, intravenous Ig (IVIG), intralipid, and TNF-α–blocking agent. Interesting results have been obtained with IVIG treatment in antiphospholipid syndrome with persistent presence of autoantibodies against beta2 glycoprotein 1, where NK cell expansion (Oliver-Minarro et al., 2009) and Th1 shift (Benagiano et al., 2017) seem to be implicated in recurrent miscarriage. It is necessary to underline that during viral infections it is not inhibition of eNK cells that is needed, but rather the right degree of activation that is of importance.

Future studies, with a large cohort of infertile idiopathic women will be necessary to elucidate the possible role of eNK cell cytotoxicity toward HHV-6A endometrial infected cells, endometrial health status and embryo implantation. Understanding mechanisms that regulate eNK cell activation will clarify their involvement in female idiopathic infertility.

## Author Contributions

EC contributed to the conception of the work and data analysis. DB, AR, VG, IS, MD, MS, and IB contributed to data collection and analysis. RM and GLM contributed to clinical samples collection. DDL contributed to data interpretation and critical revision of the article. RR contributed to the conception of the work, data acquisition and analysis, and writing the article.

## Conflict of Interest Statement

The authors declare that the research was conducted in the absence of any commercial or financial relationships that could be construed as a potential conflict of interest.
