# Supplementary figures and images for: HHV-6A Infection of Endometrial Epithelial Cells Induces Increased Endometrial NK Cell-Mediated Cytotoxicity
Source: Front Microbiol. 2017 Dec 15;8:2525. doi: 10.3389/fmicb.2017.02525 (PMC5736868; doi:10.3389/fmicb.2017.02525)

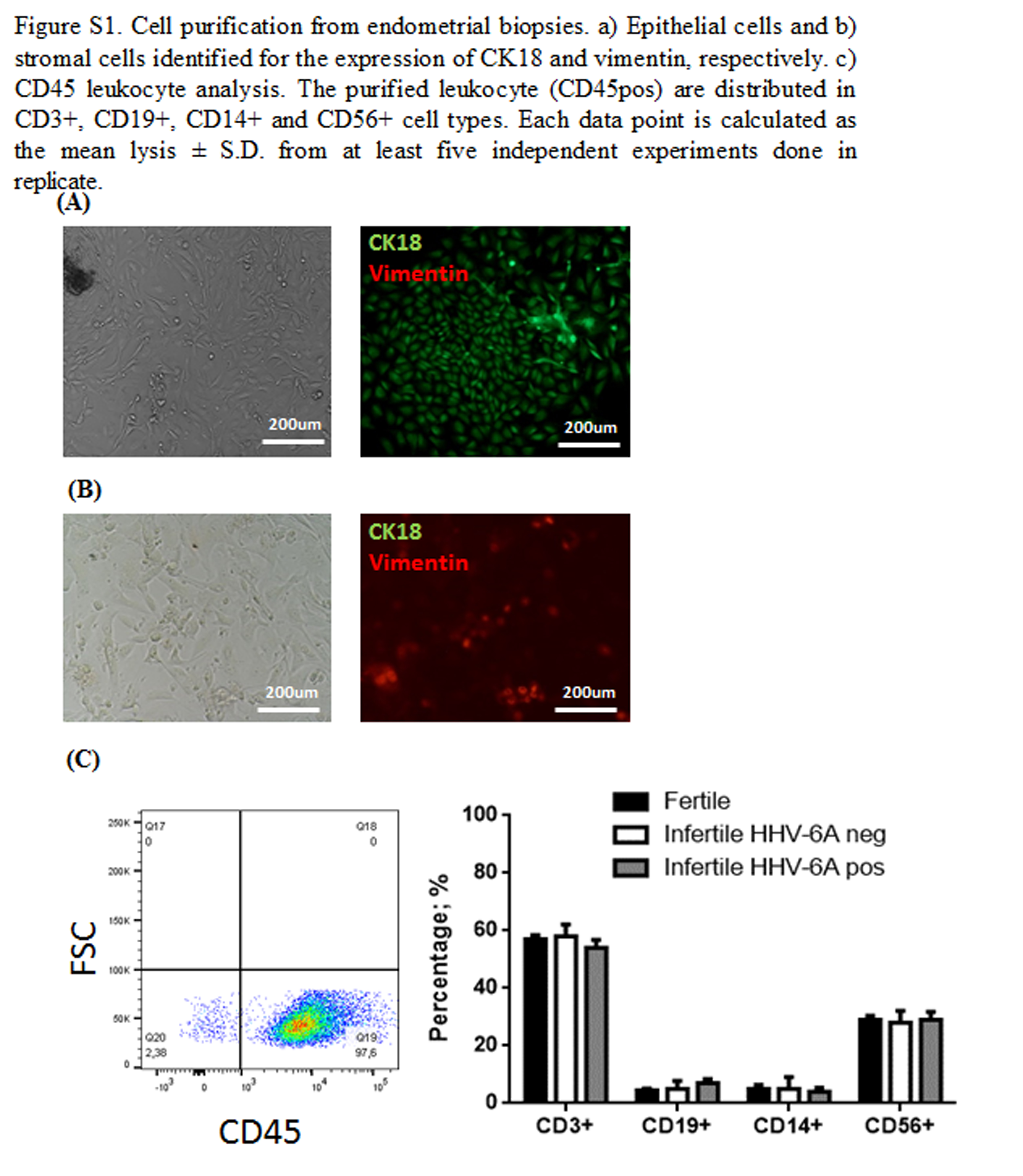

Supplement: Supplementary file 1 [file Image_1.JPEG]

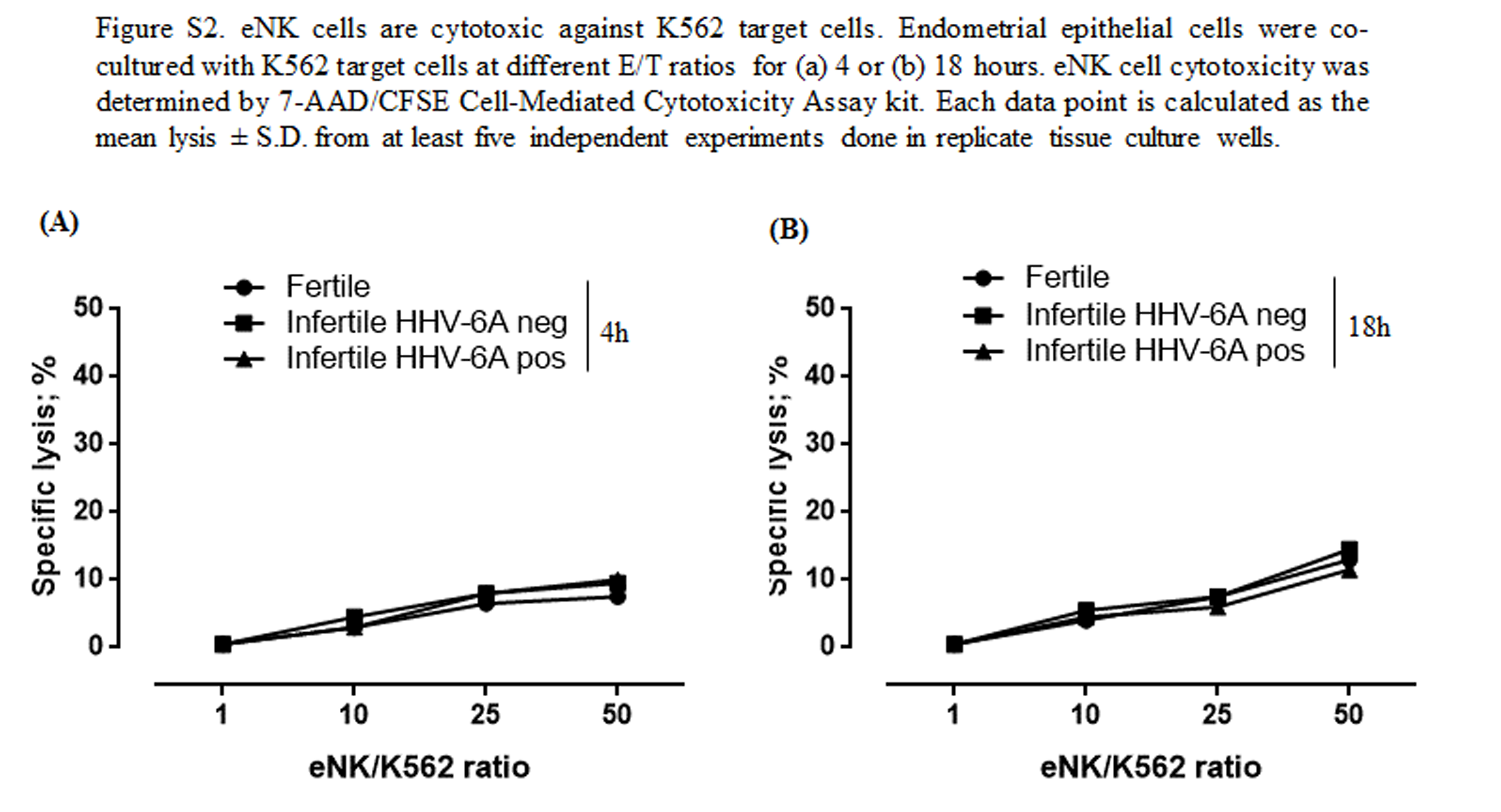

Supplement: Supplementary file 2 [file Image_2.JPEG]
